# Supplementary material for: The Effect of Adjuvant Chemotherapy on Localized Extraskeletal Osteosarcoma: A Systematic Review
Source: Cancers (Basel). 2022 May 23;14(10):2559. doi: 10.3390/cancers14102559 (PMC9139294; doi:10.3390/cancers14102559)
Supplement: Supplementary file 1 [file cancers-14-02559-s001.zip › cancers-1718072-supplementary.pdf]

Systematic Review

The Effect of Adjuvant Chemotherapy on Localized Extraskeletal Osteosarcoma: A Systematic Review

Shinji Tsukamoto [1], Andreas F. Mavrogenis [2,*], Lucia Angelelli [3], Alberto Righi [4], Giuseppe Filardo [3], Akira Kido [5], Kanya Honoki [1], Yuu Tanaka [6], Yasuhito Tanaka [1] and Costantino Errani [7]

Table S1. Literature search and study selection. (Supplementary material).

	**PubMed**	2022/3/26
#	Searched for	Results
1	Extraskeletal osteosarcoma *[tiab] OR extraosseous osteosarcoma *[tiab] OR soft tissue osteosarcoma *[tiab] OR extra-skeletal osteosarcoma *[tiab] OR extra-osseous osteosarcoma *[tiab]	458
	**Embase**	2022/3/26
Set#	Searched for	Results
S1	(TI,AB((extraskeletal* OR extraosseous* OR (soft P/0 tissue*) OR “extra-skeletal” OR “extra-osseous”) P/0 osteosarcoma*))	546°
	**Central**	2022/3/26
ID	Searched for	Results
#1	((extraskeletal* NEXT osteosarcoma*) OR (extraosseous* NEXT osteosarcoma*) OR (soft NEXT tissue* NEXT osteosarcoma*) OR (“extra-skeletal” NEXT osteosarcoma*) OR (“extra-osseous” NEXT osteosarcoma*)):ti,ab,kw	1
